# Three case reports of patients indicating the diversity of molecular and clinical features of 16p11.2 microdeletion anomaly

**DOI:** 10.1186/s12920-021-00929-8

**Published:** 2021-03-10

**Authors:** Monika Szelest, Martyna Stefaniak, Gabriela Ręka, Ilona Jaszczuk, Monika Lejman

**Affiliations:** 1 Student Scientific Society, Laboratory of Genetic Diagnostics, Medical University of Lublin, Gębali 6, 20-093 Lublin, Poland; 2 Department of Cancer Genetics With Cytogenetics Laboratory, Medical University of Lublin, Radziwiłłowska 11, 20-080 Lublin, Poland; 3 Laboratory of Genetic Diagnostics, Medical University of Lublin, A. Gębali 6, 20-093 Lublin, Poland

**Keywords:** 16p11.2, Microdeletion anomaly, Autism spectrum disorder, Microarray, MLPA

## Abstract

**Background:**

16p11.2 microdeletion is a known chromosomal anomaly associated mainly with neurocognitive developmental delay, predisposition to obesity, and variable dysmorphism. Although this deletion is relatively rare among the general population, it is one of the serious known genetic aetiologies of obesity and autism spectrum disorder.

**Case presentation:**

This study presents three cases of deletions within the 16p11.2 region. Every child had mild variable craniofacial abnormalities, hand or foot anomalies and developmental and language delays. The first proband had obesity, epilepsy, moderate intellectual disability, aphasia, motor delay, hyperinsulinism*,* and *café au lait* spots. The second proband suffered from cardiac, pulmonary, and haematological problems. The third proband had motor and language delays, bronchial asthma, and umbilical hernia. Although each patient presented some features of the syndrome, the children differed in terms of their clinical pictures. Genetic diagnosis of 16p11.2 microdeletion syndrome was made in children at different ages based on multiplex ligation probe-dependent amplification analysis and/or microarray methods.

**Conclusions:**

Our reports allow us to analyse and better understand the biology of 16p11.2 microdeletion throughout development. However, the variability of presented cases supports the alternate conclusion to this presented in available literature regarding 16p11.2 deletion, as we observed no direct cause-and-effect genotype/phenotype relationships. The reported cases indicate the key role of the interdisciplinary approach in 16p11.2 deletion diagnostics. The care of patients with this anomaly is based on regular health assessment and adjustment of nervous system development therapy.

**Supplementary Information:**

The online version contains supplementary material available at 10.1186/s12920-021-00929-8.

## Background

16p11.2 deletion syndrome is a known chromosomal aberration with an estimated prevalence of 1–5/10,000 in the general population [1]. Research based on the large ClinGen database suggests that 16p11.2 deletions are the second most commonly identified microdeletion, occurring in one of every 235 cases tested with intellectual and developmental disability [2]. Interestingly, this deletion confers susceptibility to autism spectrum disorder (ASD) in nearly 1% of people with autism and has been found in up to 0.001% of people with psychiatric disorders [3–5]. The amount and location of deleted genetic material allows distinguishing different categories of this chromosomal anomaly. However, 16p11.2 microdeletion syndrome is usually caused by a deletion of an approximately 600-kbp region containing 29 protein-coding genes [6].

Most cases of these microdeletions are not inherited, as de novo deletions are found in approximately 75% of children [7]. 16p11.2 microdeletion is mainly characterized by neurocognitive developmental delay, intellectual disability, and ASD [8]. Moreover, the phenotypic spectrum associated with this deletion is much wider and includes delays in speech or motor development, language impairment (apraxia or dysarthria), low muscle tone, hypo- or hyperreflexia, a tendency towards obesity, short stature, and hyperinsulinaemic hypoglycaemia [6, 8–10]. Patients with this chromosome anomaly present variable craniofacial abnormalities, such as macro- or microcephaly, hypertelorism, full cheeks, posterior rotated ears, downslanting palpebral fissures, deep-set eyes, ptosis, and a small nose with a broad nasal bridge [8, 11]. Changes in the fingers and toes can also be observed, mainly in the form of bilateral fifth finger clinodactyly and syndactyly of the second and third toes [12]. Moreover, 16p11.2 microdeletion is associated with dermatological alterations, for example, *café au lait* spots and sacral dimples [13]. Other clinical features of 16p11.2 deletions include anxiety disorders, attention deficit hyperactivity disorder (ADHD), and reduced fertility [14]. Moreover, the deleted fragment is particularly important in speech development and is observed in more than 70% of patients with 16p11.2 deletions [15]. The clinical picture of 16p11.2 microdeletion may vary among patients, depending on the size of the lost chromosome fragment.

In this study, we report the cases of three patients with 16p11.2 microdeletions. Although each child had the craniofacial features, hand or foot anomalies and developmental delays characteristic of 16p11.2 microdeletions, the patients differed in their overall clinical pictures. Multiplex ligation probe-dependent amplification (MLPA) analysis and microarray testing were used to confirm the cytogenetic aspect of the diagnosis. All three patients had genomic imbalances encompassing one or both recurrent regions in 16p11.2.

## Case presentation

**Fig. 1:**
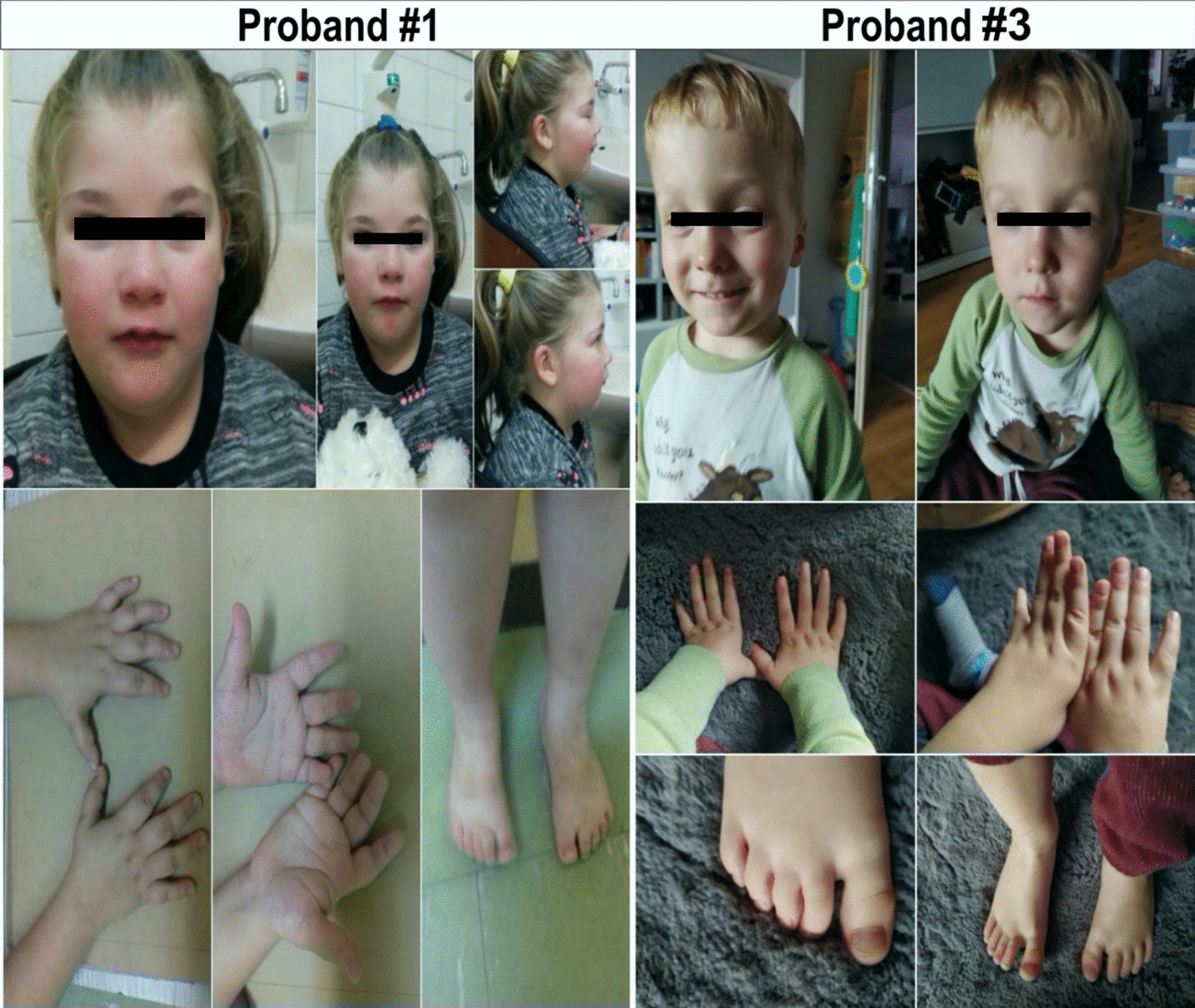
Photos of proband #1 and proband #3. Proband #1 (left side of the panel), showing some unusual facial features, such as a retracted forehead, long eyelid fissures, a wide back of the nose, a tented upper lip, a small mandible, and a retracted chin. Proband #2 (right side of the panel), exhibiting macrocephaly, protruding frontal and parietal tubers, flattened facial profile, upward oblique eyelid slanting, low and wide bridge of the nose, short nose spine, wide philtrum, receding chin, back-folded auricles, gothic palate, and small hands

**Fig. 2:**
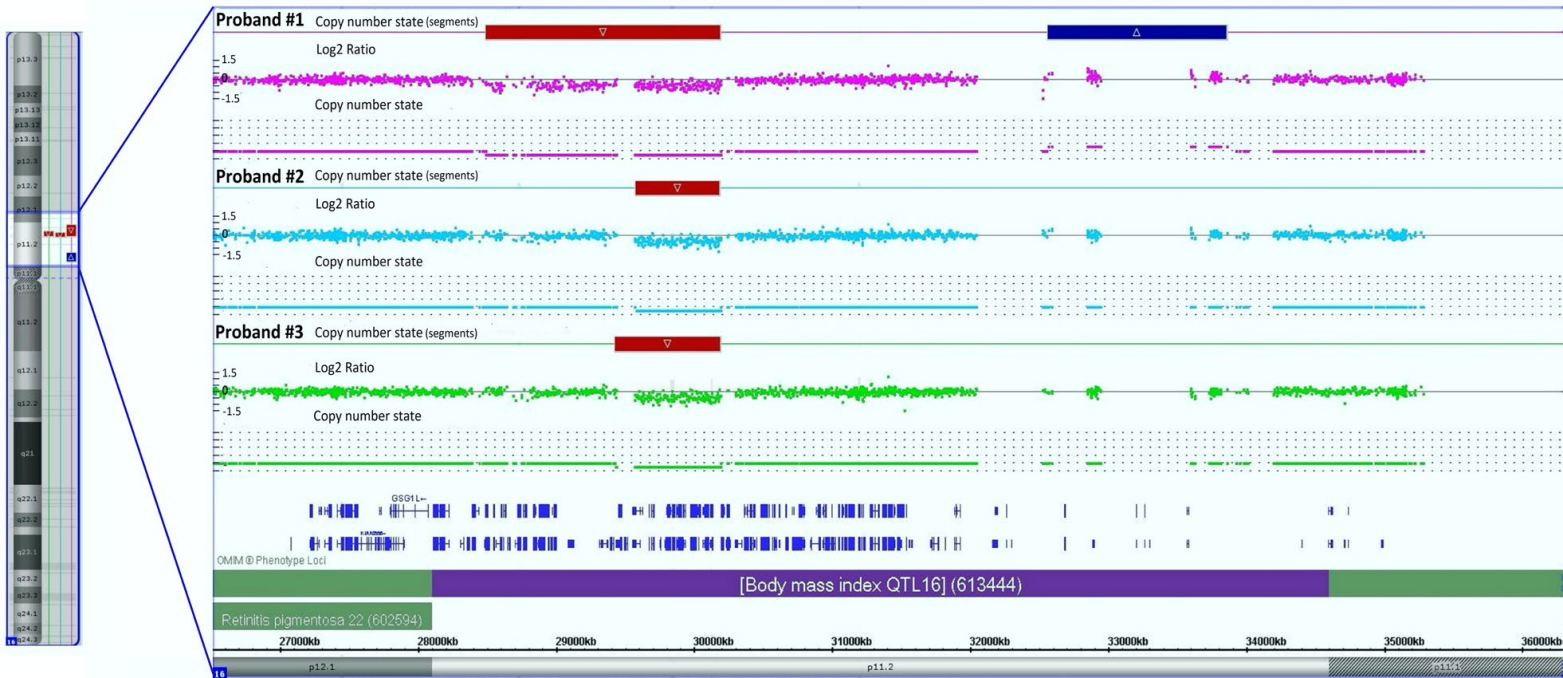
Microarray results of proband #1, proband #2, and proband #3 samples. Microarray results show genomic imbalance of deletions in the 16p11.2 chromosome region

The first described proband was born as the third child (from the fourth pregnancy, the third birth) of an unrelated couple in a family where no intellectual disabilities or congenital genetic defects were reported. The two previous deliveries were performed on time with a caesarean section. The girl's mother had one spontaneous miscarriage. During pregnancy, the proband’s mother suffered from gestational diabetes treated with a diet, arterial hypertension treated with a methyldopa preparation, and was on short-term medications for colds. The influence of teratogenic factors was not reported, and foetal ultrasound was normal. Pregnancy was resolved in the 40th week by caesarean section. The patient's body weight after birth was 3330 g, body length was 55 cm, and head circumference was 34 cm. The child's development after birth was abnormal, and due to the increased muscle tension, she received rehabilitation therapy from the age of 3 months. At 8 months of age, seizures occurred, and drug-resistant epilepsy treated with levetiracetam was diagnosed. From the age of 2, an increased appetite (hyperphagia) followed by a rapid increase in body weight led to obesity: at the age of 5.5, the girl was 137 cm tall, with a body weight of 37 kg and a body mass index (BMI) of 27 kg/m^2^ (> 95th percentile, > 2SD; calculated on the basis of percentile scales developed for the population of Polish children by Palczewska and Niedźwiedzka) [16]. The child's development was assessed as delayed, with moderate intellectual disability and impaired speech development. The patient was also diagnosed with binocular hyperopia (+ 1.0 dioptre), and she wore corrective glasses. At the age of 5.5 years, the girl was admitted to the Department of Pediatrics, Endocrinology and Diabetology of Children's University Hospital in Lublin owing to obesity. Laboratory deviations, such as hyperinsulinism, based on the oral glucose tolerance test (OGTT), relatively low levels of morning cortisol in the absence of clinical symptoms, and dyslipidaemia, were found in the study. Abdominal ultrasonography performed in May 2018 showed no abnormalities, except for slight liver and spleen enlargement. The examination showed bitemporal narrowing of the skull, short hands and feet, hand and foot brachydactyly, and partial syndactyly of the second and third toes. The study also revealed other anomalies, such as a narrow forehead, a flattened facial profile, upslanting palpebral fissures and their almond shape, hypotelorism, a short column of the nose, down-turned corners of the mouth, and a highly arched palate (Fig. 1). Due to the suspicion of Prader–Willi syndrome, a methylation-specific multiplex ligation-dependent probe amplification (MS-MLPA) test was performed (ME-028-C1 Prader–Willi/Angelman, MRC Holland, Amsterdam, Netherlands). Both the methylation and genomic profiles were normal. Genetic tests were extended by use of the CytoScan 750 K array (750,000 oligonucleotide probes and 200,000 probes identifying a single-nucleotide polymorphism (SNP) (Applied Biosystems, Thermo Fisher Scientific, Waltham, MA, USA). A microarray revealed a deletion including 72 genes of region 16p11.2 (28490479_30190029), spanning 1699.55 kbp (Fig. 2). The girl, due to an abnormal psychomotor development, intellectual disability and speech development disorders, is under constant psychological, speech therapy and pedagogical care. Currently, due to increasing behavioral disorders in the form of behavioral rigidity, difficulties in adapting to new situations and in verbal communication, a selective diet, and tantrums, the diagnosis of early childhood autism has been established.

The second patient was born as the first child of a mother who did not have a miscarriage, and no genetic diseases were present in the family history. The baby was born on time by means of a caesarean section in favourable general condition with a birth weight of 2930 g. The course of pregnancy was complicated by arterial hypertension and urinary tract infections of the mother. The test for β-haemolytic streptococci in group B was positive. On the second day of the girl's life, respiratory disorders—tachypnoea and decreases in saturation up to 80%—were reported; therefore, congenital pneumonia was diagnosed. Additional examinations revealed right heart dilatation, features of pulmonary hypertension, and a patent ductus arteriosus with right-left flow. During hospitalization in the Department of Hematology, Oncology and Transplantology Children's Hospital, an improvement in haemodynamic parameters was observed. Control echocardiography showed a foramen ovale (FO) and detrended fluctuation analysis (DFA) with left–right flow and aortic obstruction. Ultrasound examination of the central nervous system (CNS) revealed features of first-degree ventricular bleeding. The girl received haematological and transfusion consultations based on thrombocytopenia. These consultations indicated thrombosis at the site of central venous catheter implantation. Neurological consultation did not reveal features of neurological syndrome. Physical examination of the patient uncovered a number of anomalies, such as a retracted forehead, long eyelid fissures, a wide back of the nose, a tented upper lip, a small mandible, a retracted chin, and clinodactyly of the fifth fingers. Owing to the presence of heart defects and craniofacial features and the suspicion of immune deficiency due to congenital pneumonia, a deletion in 22q11.2 (DiGeorge syndrome) was suspected. DiGeorge syndrome was excluded by MLPA using the SALSA MLPA kit (P245-B1 Microdeletion Syndromes-1 and P297-C1 Microdeletion Syndromes-2). The MLPA probe sizes, chromosomal positions, and sequences are provided in Additional file 1: Table S1. MLPA P297-C1 analyses indicated a genome imbalance of deletions in the 16p11.2 chromosome region, including the *HIRIP3* (OMIM *603365), *DOC2A* (OMIM *604567), *MAZ* (OMIM *600999), *MAPK3* (OMIM *601795), and *MVP* (OMIM *605088) genes (Additional file 2: Fig. S1, A, D). The molecular karyotype confirmed a deletion in chromosome 16, region 16p11.2 (29580020_30190029) spanning 610 kbp and including 31 genes (Fig. 2). The psychomotor development of the second patient at the of 11 months is not delayed in the opinion of the pediatric neurologist. Due to the risk of the motor development disorders, speech development delays, and the risk of autism spectrum disorders, described in the 16p11.2 deletion, the patient is under the constant care of a psychologist, physiotherapists and pediatric neurologist.

The third proband was a boy abandoned by his mother in a hospital during hospitalization owing to infection at the age of two weeks. He was born in the 36th week of pregnancy, with a body weight of 2680 g, a body length of 51 cm and a head circumference of 33 cm, as the second child of a mother who had not previously had a miscarriage. There were no detailed data on the family medical history or on the course of pregnancy. He was in a foster family for 1.5 years and had been living in an adoptive family for three months. At the age of 20 months, the boy was admitted to the Pediatric Pulmonary and Rheumatology Department because of another asthma exacerbation. The boy presented developmental and language delays. He started sitting at the age of 11 months and walking at the age of 19 months. Physical examination demonstrated mild variable anomalies, including macrocephaly, protruding frontal and parietal tubers, a flattened facial profile, upslanting palpebral fissures, a low-set and wide nasal bridge, a short nasal root, a wide philtrum, a receding chin, back-folded auricles, a highly arched palate, small hands, adducted feet, and umbilical hernia (Fig. 1). Molecular tests were performed to search for the causes of the patient’s neurodevelopmental problems. The microarray analysis showed a genome imbalance spanning 761 kbp, including 44 genes in region 16p11.2 (29428531_30190029) (Fig. 2). The motor development of the third patient at the age of 3 years does not show significant abnormalities or delays in neurological assessment. The greatest neurodevelopmental problem, in the opinion of psychological and speech therapists, is incorrect and delayed speech development. The boy currently uses only single short words. However, he does not show any characteristic features that might suggest an ASD. Due to the varus position of the feet and the resulting gait disturbance, he is under the care of a physiotherapist. The clinical findings in our patients are summarized in Table 1.

**Table 1:** Clinical features of patients with 16p11.2 microdeletion syndrome

	Proband #1	Proband #2	Proband #3
Sex	Female	Female	Male
Age at diagnosis (first admission due to other symptoms)	5.5 years	1 months	2 years
Gestational age (weeks)	40	40	36
Birth weight (g)	3330	2930	2680
Weight (kg)	37	Not available	12.5
Head circumference after birth (cm)	34	Not available	33
Cognitive/behavioural developmental differences	Moderate intellectual disability, behavioural disorders—obstinacy, rebelliousness, episodes of eating inedible things, suspected ASD	Undeterminable due to young age	Motor developmental delay
Speech delay	Yes motor aphasia	Undeterminable due to young age	Yes
Motor delay	Yes	Undeterminable due to young age	Sitting at age of 11 months, walking at age of 19 months
Craniofacial dysmorphic features	Macrocephaly Narrow forehead Hypotelorism Flattened face profile Retracted chin Upslanting palpebral Fissures Almond-shaped palpebral fissures Short nasal bridge Down-turned corners of the mouth High arched palate Thickened helix of auricles	Retracted forehead Long palpebral fissures Wide nasal bridge Tenuous upper lip Small mandible Retracted chin	Macrocephaly Protruding frontal and parietal tubers Flattened face profile Oblique, narrow palpebral fissures Low-set and wide, short nasal bridge Wide philtrum Small retracted chin Auricles rotated backwards High arched palate Oblique occiput Thickened frontal seam
Brain abnormalities	EEG—small localized temporal lesions	Features of first-degree intraventricular hemorrhage	Not present
Muscle tone disorders	Hypotonia	Not present	Hypotonia
Hand /foot abnormalities	Short hands and feet, hands and feet brachydactyly, partial syndactyly of the toes 2nd and 3rd toes, knock-knees	Clinodactyly of 5th fingers	Small hands, adducted feet
Seizures	Drug-resistant epilepsy, the patient treated with levetiracetam	Not present	Not present
Cardiac abnormalities	Not present	Right heart dilatation, features of pulmonary hypertension, patent ductus arteriosus with left–right flow, foramen ovale	Not present
Other	Severe obesity, increased but selective appetite, 2 *café au lait* spots—5–6 mm in diameter, squint, tooth decay, hyperinsulinism in oral glucose tolerance test, relatively low morning cortisol level with the absence of clinical symptoms, dyslipidemia, slight liver and spleen enlargement, nose—*Moraxella catarrhalis*	Breathing problems due to congenital pneumonia, thrombocytopenia, transfusiological suspicion of thrombosis at the central venous catheter implantation place	Bronchial asthma, umbilical hernia
Prenatal interview	Pregnancy complicated by maternal hypertension	Pregnancy complicated by hypertension and maternal urinary tract infections, a history of positive test for group B β-hemolytic streptococci	Mother's nicotinism
Family history of deletions	Negative family history	Negative family history	Not available due to adoption

The deletions in the 16p11.2 region found in our three patients are consistent with known contiguous gene deletion in the region of 16p11.2 (OMIM #611913). MLPA tests were also performed on samples from the parents of the first and second children to determine carrier status. However, the MLPA test results for both the mothers and fathers of the first and second patients did not reveal carrier status (Additional file 2: Fig. S1, A, D). Upon discharge, periodic check-ins with an outpatient clinic, regular follow-ups with a clinical geneticist, consistent individualized neurological and developmental treatments, appropriate diets and regular physical activity were recommended to the patients and their families.

All deleted genes are listed in Table 2.

**Table 2:** Table showing deleted genes in the 16p.11 region in three analysed probands, according to the microarray results

	Size of deleted region (kbp)	Result of microarray ISCN 2016	Deleted genes	Common deleted genes
Proband #1	1699.55	arr[GrCh37] 16p11.2(28490479_30190029) × 1	***CLN3****, APOBR, IL27, NUPR1, SGF29, SULT1A2, SULT1A1, NPIPB8, EIF3C, EIF3CL, MIR6862-1, MIR6862-2, NPIPB9, * ***ATXN2L*** *, TUFM, MIR4721, * ***SH2B1,*** ***ATP2A1, ATP2A1-AS1*** *, RABEP2, CD19, NFATC2IP, MIR4517, SPNS1, LAT, RRN3P2, SNX29P2, NPIPB11, SMG1P6, BOLA2-SMG1P6, LOC606724, BOLA2, BOLA2B, SLX1B, SLX1A, SLX1A-SULT1A3, SLX1B-SULT1A4, SULT1A4, SULT1A3, LOC388242, LOC613038, SMG1P2, MIR3680-1, MIR3680-2, SPN, QPRT, C16orf54, ZG16, KIF22, MAZ, PRRT2, PAGR1, MVP, CDIPT, CDIPT-AS1, SEZ6L2, ASPHD1, KCTD13, TMEM219, TAOK2, HIRIP3, INO80E, DOC2A, C16orf92, FAM57B, ALDOA, PPP4C, TBX6, YPEL3, LOC101928595, GDPD3, MAPK3*	*SMG1P2, MIR3680-1, MIR3680-2, SPN, QPRT, C16orf54, ZG16, KIF22, MAZ, PRRT2, PAGR1,* *** MVP*** *, CDIPT, CDIPT-AS1, SEZ6L2, ASPHD1, * ***KCTD13*** *, TMEM219, * ***TAOK2*** *, HIRIP3, INO80E, * ***DOC2A*** *, C16orf92, FAM57B, * ***ALDOA*** *, PPP4C, * ***TBX6*** *, YPEL3, LOC101928595, GDPD3,* *** MAPK3***
Proband #2	610	arr[GrCh37] 16p11.2(29580020_30190029) × 1	*SMG1P2, MIR3680-1, MIR3680-2, SPN, QPRT, C16orf54, ZG16, KIF22, MAZ, PRRT2, PAGR1, MVP, CDIPT, CDIPT-AS1, SEZ6L2, ASPHD1, KCTD13, TMEM219, TAOK2, HIRIP3, INO80E, DOC2A, C16orf92, FAM57B, ALDOA, PPP4C, TBX6, YPEL3, LOC101928595, GDPD3, MAPK3*	
Proband #3	761	arr[GrCh37] 16p11.2(29428531_30190029) × 1	*SMG1P6, BOLA2-SMG1P6, LOC606724, BOLA2, BOLA2B, SLX1B, SLX1A, SLX1A-SULT1A3, SLX1B-SULT1A4, SULT1A4, SULT1A3, LOC388242, LOC613038, SMG1P2, MIR3680-1, MIR3680-2, SPN, QPRT, C16orf54, ZG16, KIF22, MAZ, PRRT2, PAGR1, MVP, CDIPT, CDIPT-AS1, SEZ6L2, ASPHD1, KCTD13, TMEM219, TAOK2, HIRIP3, INO80E, DOC2A, C16orf92, FAM57B, ALDOA, PPP4C, TBX6, YPEL3, LOC101928595, GDPD3, MAPK3*	

## Discussion and conclusions

In our study, each described patient revealed some features of 16p11.2 microdeletion anomaly according to previously reported data [3–5, 17]. Furthermore, in 2 of the 3 presented cases, the 16p11.2 deletion occurred de novo. In the case of the third patient, it was not possible to examine the parents because the patient was adopted. Despite its significant incidence in the general population, 16p11.2 microdeletion anomaly deletion has a variable clinical picture. There are no strict genotype–phenotype correlations. However, several genes within the 16p11.2 region (e.g., *TAOK2*) might interact with each other via common signalling pathways, thus affecting single gene phenotypes [18]. Further studies are needed to identify these candidate genes to determine their role in neuropsychiatric disorders. In our patients, we could not identify the permanent common features among all patients, except the small receding chin. The phenotypic features were most similar in the first and third patients: macrocephaly, upslanting palpebral fissures and a highly arched palate. In addition, the neurodevelopmental picture was dominated by psychomotor retardation in the first 2 years of life and speech development disorders (the first and third patients). Pizzo at el. reported that 16p11.2 deletion carriers could have the burden of rare deleterious mutations within genes in the genetic background correlated with the variability of IQ scores and head circumference [19].

Although deletions within the 16p11.2 region are associated with a wide spectrum of symptoms, several studies have provided a comprehensive characterization of patients with this chromosomal imbalance [17, 20, 21]. These reports have allowed us to analyse and better understand the biology of 16p11.2 microdeletion throughout development. To date, a few hypotheses have been proposed to characterize the association between a deletion within the 16p11.2 region and its clinical manifestation [18, 22, 23]. As outlined above, neuropsychiatric phenotypes might result from altered cell signalling. On the other hand, Shinawi et al. reported a dose effect of 16p11.2 copy number on the different clinical results, proposing the occurrence of dosage-sensitive genes within the region [22, 23].

However, the molecular mechanisms underlying the clinical features presented by patients with 16p11.2 microdeletions remain unclear and need additional investigation. Taking into consideration the variability of the phenotype in presented cases and relatively low penetrance of copy number variations (CNVs) regarding 16p11.2 deletions, we surmise that the outcome might act through the “two-hit hypothesis”. Therefore, the genetic background might contribute to phenotypic heterogeneity [19]. Moreover, the clinical manifestation of the 16p11.2 deletions is modulated by additional variants. Thus, accurate genetic diagnosis of patients with 16p11.2 deletions will require the comprehensive assessment of the genetic background, which is associated with the variability and severity of clinical features in patients with the 16p11.2 anomaly [19].

The clinical picture of all three presented cases highlights the complexity of CNV-related neurodevelopmental features. These data suggest that individual genes within changeable expressed CNV regions are not responsible for the diversity of neuropsychiatric phenotypic features, and establishing whether the phenotype of a single gene completely displays the variable phenotypes of the whole CNV region is impossible. Therefore, it seems to be crucial to determine the interaction-based pattern of genes within specific CNVs to unravel their impact on important signalling pathways and, in turn, variable clinical features [18]. Since the molecular basis of the 16p11.2 region is still limited, early identification of imbalances within this region is vital to ensure appropriate treatment and psychological support.

Regarding molecular differences among the presented cases, we analysed the reported features associated with genes within a deleted chromosomal region. The most common deletion region is proximal breakpoint 4–5 (BP4-BP5), which is associated with microdeletion (OMIM#611913). Abnormalities in copy number are associated with neuropsychiatric phenotypes, growth abnormalities, skeletal abnormalities and other, less frequent congenital anomalies. The distal BP2–BP3 16p11.2 region is another frequent region spanning approximately 220 kb. Phenotypes associated with microdeletion include obesity, generalized overgrowth, global developmental delay, delayed speech and language development, ASD, seizures, ADHD, and less often congenital anomalies of other organ systems [12]. It is worth mentioning that Proband 1 presented the largest deletion that contained both the BP2-BP3 and BP4-BP5 recurrent regions. Furthermore, the deletion featured, among others*,* the *CLN3* gene. The patient's symptoms included epilepsy, diagnosed in the 8th month of life, and binocular hyperopia diagnosed in the 7th year of life. Recessive *CLN3* mutations are the cause of neurodegenerative disorders, characterized by progressive vision failure and seizures. The set of symptoms associated with *CLN3* mutations is described as juvenile neuronal ceroid lipofuscinosis (JNCL). In the presence of the *CLN3* mutation and hemizygous 16p11.2 deletion, JNCL symptoms are more pronounced than the symptoms of the 16p11.2 microdeletion [24, 25]. In summary, phenotypic variability among patients with 16p11.2 deletions may also be associated with the expression of recessive mutations in the non-deleted homologue [26].

One of the significant symptoms of 16p11.2 deletions is the tendency towards obesity. The *SH2B1* gene, with functions that are associated with the regulation of leptin and insulin signalling, is another gene within the 16p11.2 microdeletion region in the first patient. *SH2B1* deletion is associated with an increased risk of obesity as a result of hyperphagia and insulin resistance [27, 28]. Duan et al. described that insulin signalling in skeletal muscle, liver, and fat can be impaired by systemic deletions in the mouse *SH2B* gene [27]. Hyperinsulinism in the OGTT and dyslipidaemia (reduced HDL levels) were observed in the first patient during hospitalization. Moreover, deletions in the 16p11.2 *SH2B1*-containing region are associated with developmental delays, such as motor and language delays [12]. These symptoms correlate with those observed in the first proband. Similar neurodevelopmental disorders occurred in the third patient however, without the deletion of the *SH2B1* gene. This could be explained by the loss of function of *HIRIP3*, *KIF22*, and *PPP4C* which may lead to spontaneous movement defects or touch disorders [29].

Although 16p11.2 deletions are linked to ASD, in our report, one out of three patients exhibited autism features; what was finally confirmed by psychological diagnosis [18]. 16p11.2 deletions influence the formation of brain structures, as neuroimaging investigations have indicated structural abnormalities, mainly affecting the grey matter [30]. The observed structural changes in the cerebral cortex are considered to be the causes of a decreased intellectual quotient and a higher risk of ASD. Pucilowska et al. indicated the probable role of the *MAPK3* gene in the synaptic signalling pathway, which is important in the learning process [31]. Increased neuronal progenitor proliferation and the dysregulation of apoptosis in neurons were observed in mice and zebrafish with 16p11.2 deletions [31, 32]. Studies by Bertero et al. suggested that neurodevelopmental disorders and abnormal socio-cognitive function are the result of abnormal long-range prefrontal synchronization in the brain structure [33]. Wu et al. identified heterozygous *TBX6* null mutations that were related to congenital scoliosis in a Han Chinese population. Furthermore, these null alleles included CNVs (12 instances of a 16p11.2 deletion affecting *TBX6)* [34]*.* Studies have also revealed critical roles of *MAPK3*, *KCTD13, MVP,* and *TAOK2* in the growth and proliferation of progenitor cells and in neurite morphogenesis in ASD [31, 32]. Richter et al. reported that alterations within the *TAOK2*, *HIRIP3*, and *DOCA2* genes are associated with ASD pathogenesis, as they encode proteins responsible for proper development of nerve tissue, especially synaptic connections. In particular, the *TAOK2* gene is regarded as a new gene factor in the development of neurodevelopmental disorders [35]. The above-mentioned genes were identified as deleted in all probands. Jensen et al. reported *TAOK2, MVP, ALDOA, DOC2A* (alterations of these genes was observed in all our probands) and *ATXN2L, ATP2A, SH2B1* in distal 16p11.2 regions (detected in the first proband only) as candidate genes that are responsible for developmental disorders with genome-wide metrics of pathogenicity, including measurements of haploinsufficiency [18].

However, over the period of the report, 250 patients with phenotypic characteristic that appear to overlap with 3 reported cases (neurodevelopmental disorders, impaired speech development, psychomotor delay etc.) and 150 patients with neurodevelopmental delay and ASD or ADHD were tested. These numbers confirm that (similar with other case series) we are dealing with case selection bias. Moreover, a small proportion of tests showed a 16p11.2 deletion, thus raising additional concern regarding the validity of genotype/phenotype correlation. Thus, it might be relevant to perform additional genetic analyses, such as whole exome sequencing, in order to identify novel pathogenic variants, contributing the phenotype of patients with the 16p11.2 deletion. The variety of clinical phenotypes among patients with 16p11.2 aberrations makes it difficult to interprete their examination results. Regarding adults with the 16p11.2 deletion, it was reported that several phenotypes are associated with this anomaly, such as a high incidence of diabetes, hypertension and osteoarthritis, suggesting the requirement for systematic medical observation. Although carriers of 16p11.2 deletion might present subtle cognitive distortions, it might affect their ability to earn for a living and educational attainment in adulthood. Therefore, our study highlights the importance of routine genetic testing in patients with speech delay, ASD, cognitive impairment, and other symptoms connected with neurodevelopmental disorders. Furthermore, it is crucial to diagnose patients with the 16p11.2 microdeletions at younger age, as it provides early clinical monitoring and psychical care of patients; and prevents from long-term sequalae.

The reported cases indicate a key role of an interdisciplinary approach in the diagnosis and care of patients with 16p11.2 deletion. The diagnosis of 16p11.2 microdeletion is made at different ages based on molecular tests, such as MLPA and microarray analyses. However, the variability of presented cases supports the alternate conclusion to this presented in available literature regarding 16p11.2 deletion, as we observed no direct cause-and-effect genotype/phenotype relationships. The clinical observations do not allow unambiguous determination of the phenotypic features characteristic of 16p11.2 deletions. Therefore, further studies are needed in order to determine the actual frequency of this aberration and provide a comprehensive characteristic of clinical features regarding 16p11.2 deletions.

## Supplementary Information

**Additional file 1: Table S1**. Examples of microdeletion syndromes in MLPA (P245-B1) (A) and MLPA (P297-C1) (B).

**Additional file 2: Fig. S1**. MLPA results for proband #1 and her parents. MLPA scatter plots for DNA of proband #1 (A) and her parents (B, C) are presented. Test probes (green points) are within the normal range (located between green lines) for DNA of the patient’s parents, while test probes (red points) appear lowered for DNA of proband #1. The table (D) notes the copy number of each used probe. Regarding proband #2 and proband #3, the MLPA for both the mother and father did not indicate carrier status.

## Data Availability

The datasets generated and/or analysed during the current study are available in the Gene Expression Omnibus (GEO) repository: https://www.ncbi.nlm.nih.gov/geo/query/acc.cgi?acc=GSE159129

## Abbreviations

ASD: Autism spectrum disorder
MLPA: Multiplex ligation probe-dependent amplification
BMI: Body mass index
OGTT: Oral glucose tolerance test
FO: Foramen ovale
DFA: Detrended fluctuation analysis
PB: Peripheral blood
CNA: Copy number of altered regions
CNS: Copy number states
HMM: Hidden Markov Model
CNV: Copy number variation
OMIM: Online mendelian inheritance in man
ADHD: Attention deficit hyperactivity disorder
JNCL: Juvenile neuronal ceroid-lipofuscinosis
