# Supplementary material for: Three case reports of patients indicating the diversity of molecular and clinical features of 16p11.2 microdeletion anomaly
Source: BMC Med Genomics. 2021 Mar 10;14:76. doi: 10.1186/s12920-021-00929-8 (PMC7945342; doi:10.1186/s12920-021-00929-8)
Supplement: Supplementary file 1 — Additional file 1: Table S1. Examples of microdeletion syndromes in MLPA (P245-B1) (A) and MLPA (P297-C1) (B). [file 12920_2021_929_MOESM1_ESM.docx]

Table 1A.

| Salsa MLPA P245-B1 microdeletion syndromes-2 probemix | | |
| --- | --- | --- |
| Gene | locus | microdeletion syndrome |
| TNFRSF4, GABRD, GNB1 | 1p36.33 | microdeletion 1p36 |
| REL, PEX13 | 2p16.1 | microdeletion 2p16.1 (moderate to severe mental retardation) |
| MBD5 | 2q23.1 | microdeletion 2q23.1 (intellectual disability, epilepsy, autism features) |
| SATB2 | 2q33.1 | microdeletion 2q33.1 (severe mental retardation, growth retardation, tooth abnormalities) |
| DLG1 | 3q29 | microdeletion 3q29 (mild to moderate mental retardation) |
| LETM1, WHSC1 | 4p16.3 | Wolf-Hirschhorn syndrome |
| SEMA5A, TERT | 5p15.31 | Cri du Chat syndrome |
| NSD1 | 5q35.3 | Sotos syndrome |
| ELN | 7q11.23 | Williams-Beuren syndrome |
| TRPS1 | 8q23.3 | Langer-Giedion syndrome |
| EXT1 | 8q24.11 | Langer-Giedion syndrome |
| PTCH1, FANCC | 9q22.32 | microdeletion 9q22.3 (mental retardation, overgrowth, trigonocephaly) |
| GATA3 | 10p14 | DiGeorge region 2 (10p1) |
| UBE3A, SNRPN | 15q11.2 | Prade-Willi-Angelman syndrome |
| SEMA7A | 15q24 | microdeletion 15q24 (mental retardation, growth retardation) |
| CYP1A1 | 15q24.1 | microdeletion 15q24 (mental retardation, growth retardation) |
| CREBBP | 16p13.3 | Rubinstein-Taybi syndrome |
| LRRC48, LLGL1, RAI1 | 17p11.2 | Smith-Magenis syndrome |
| NF1 | 17q11.2 | microdeletion NF1 |
| PAFAH1B1 | 17p13.3 | Miller-Dieker syndrome |
| MAPT, KANSL1 | 17q21.31 | microdeletion 17q21.31 (severe psychomotor developmental delay, facial dysmorphism, microcephaly) |
| CLDN5, GP1BB, SNAP29 | 22q11.21 | DiGeorge syndrome |
| PPIL2, RTDR1 | 22q11.21 | distal 22q11 syndrome |
| SHANK3, RABL2B | 22q13.33 | Phelan-McDermid syndrome |

Table 1B.

| Salsa MLPA P297-C1 microdeletion syndromes-2 probemix | | |
| --- | --- | --- |
| Gene | locus | microdeletion syndrome |
| PDE4DIP, CD160, HFE2, PEX11B | 1q21.1 | thrombocytopenia-absent radius (TAR) syndrome |
| FMO5, BCL9, GJA8, PRKAB2, ACP6 | 1q21.1 | others than TAR syndrome |
| DLG1, PAK2 | 3q29 | microdeletion 3q29 (mild to moderate mental retardation) |
| CNTNAP2 | 7q36.1 | microdeletion 7q36.1 syndrome (associated with autism and schizophrenia) |
| ASUN, PPFIBP1 | 12p11.23 | microdeletion 12p11.23 (associated with schizophrenia) |
| TJP1 | 15q13.1 | microdeletion 15q13.1 |
| FAN1 | 15q13.2 | microdeletion 15q13.2 |
| CHRNA7, KLF13, TRPM1, SCG5 | 15q13.3 | microdeletion 15q13.3 (mental retardation, epilepsy, variable facial and digital dysmorphisms) |
| PML | 15q24.1 | microdeletion 15q24.1(mental retardation, growth retardation) |
| HIRIP3, DOC2A, MAZ, MAPK3, MVP | 16p11.2 | microdeletion 16p11.2 (developmental delay, associated with autism and obesity) |
| PALB2 | 16p12.1 | microdeletion 16p12.1 (developmental delay) |
| IL21R | 16p12.2 | microdeletion 16p12.2 (developmental delay) |
| AATF, HNF1B, LHX1 | 17q12 | microdeletion 17q12 (mental retardation, congenital renal abnormalities, diabetes of the young type 5) |
| TCF4 | 18q21.2 | Pitt-Hopkins syndrome |
| PAK7 | 20p12.2 | microdeletion 20p11.2 (associated with schizophrenia) |
