# Supplementary figures and images for: Three case reports of patients indicating the diversity of molecular and clinical features of 16p11.2 microdeletion anomaly
Source: BMC Med Genomics. 2021 Mar 10;14:76. doi: 10.1186/s12920-021-00929-8 (PMC7945342; doi:10.1186/s12920-021-00929-8)

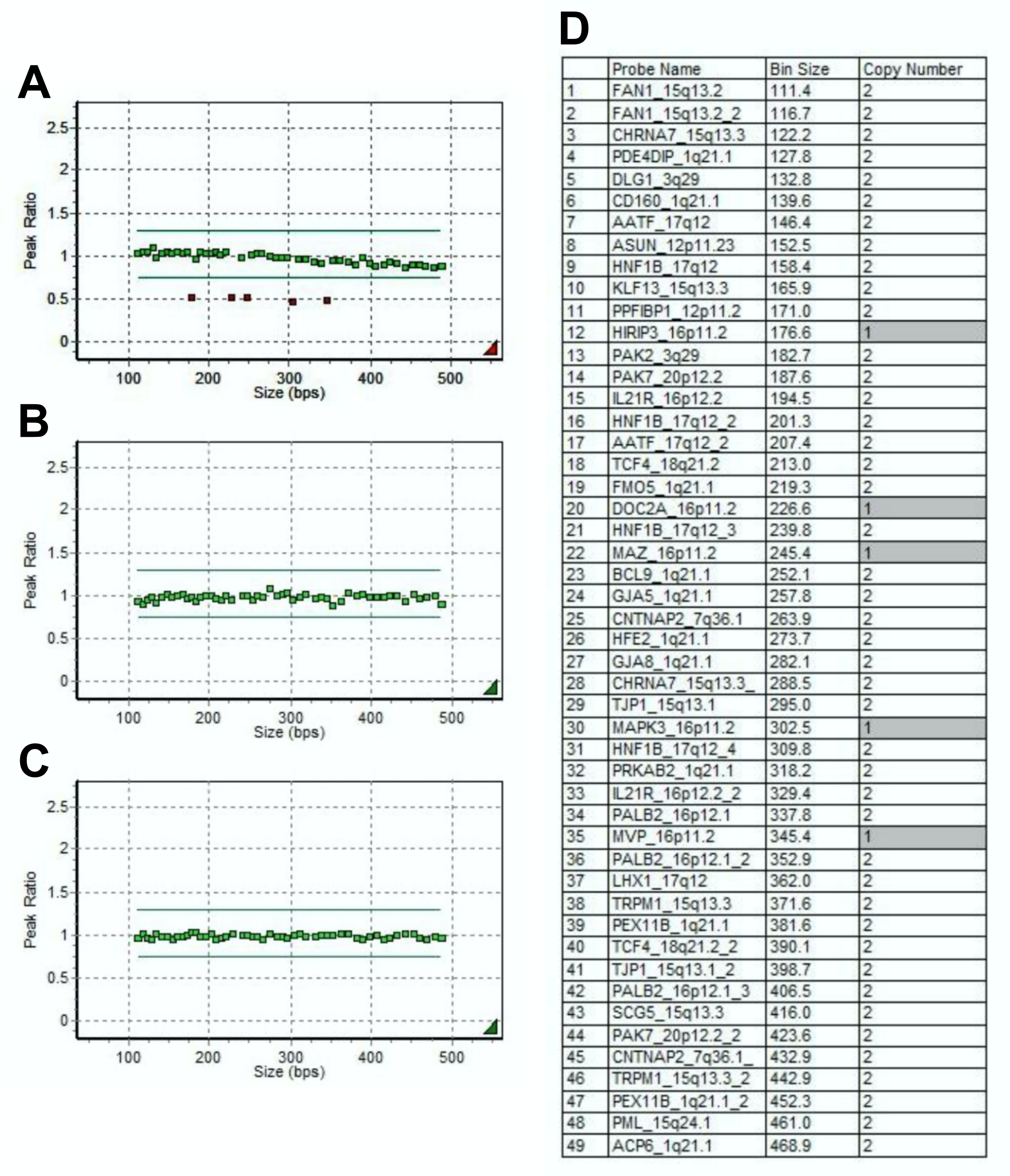

Supplement: Supplementary file 2 — Additional file 2: Fig. S1. MLPA results for proband #1 and her parents. MLPA scatter plots for DNA of proband #1 (A) and her parents (B, C) are presented. Test probes (green points) are within the normal range (located between green lines) for DNA of the patient’s parents, while test probes (red points) appear lowered for DNA of proband #1. The table (D) notes the copy number of each used probe. Regarding proband #2 and proband #3, the MLPA for both the mother and father did not indicate carrier status. [file 12920_2021_929_MOESM2_ESM.jpg]
